# Cost-effectiveness of expanding childhood routine immunization against *Neisseria meningitidis* serogroups C, W and Y with a quadrivalent conjugate vaccine in the African meningitis belt

**DOI:** 10.1371/journal.pone.0188595

**Published:** 2017-11-30

**Authors:** Andreas Kuznik, Garba Iliyasu, Mohammed Lamorde, Mustapha Mahmud, Baba M. Musa, Ibrahim Nashabaru, Stephen Obaro, Idris Mohammed, Abdulrazaq G. Habib

**Affiliations:** 1 Regeneron Pharmaceuticals, Tarrytown, NY, United States of America; 2 Infectious & Tropical Diseases Unit, College of Health Sciences, Bayero University, Kano, Nigeria; 3 Infectious Diseases Institute, Makerere University College of Health Sciences, Kampala, Uganda; 4 National Immunization Technical Advisory Group, National Primary Health Care Development Agency, Abuja, Nigeria; 5 Division of Pediatric Infectious Disease, University of Nebraska Medical Center, Omaha Nebraska, United States of America; 6 Infectious Diseases Unit, Department of Medicine, Federal Teaching Hospital, Gombe, Nigeria; RIVM, NETHERLANDS

## Abstract

**Background:**

*Neisseria meningitidis* constitutes a major public health problem among countries in the African meningitis belt. Following regional vaccination campaigns for serogroup A and subsequent increases in protection against this serogroup, non-A serogroups such as C and W now pose significant epidemic threats, particularly in young children.

**Objective:**

To evaluate the cost-effectiveness of broadening coverage from conjugate serogroup A to quadrivalent ACWY vaccination.

**Methods:**

We developed a 40-year Markov state transition model with annual cycles to simulate costs and clinical outcomes in children aged 1 to 10 in the 26 countries of the African meningitis belt. The incidence of CWY meningitis cases among an unvaccinated population was held constant at inter-epidemic rates of 50 per 100,000/year and 150 per 100,000/year. The country-specific cost and probability of access to meningitis care, vaccine efficacy, the mortality risk among treated and untreated meningitis cases, the risk of clinical sequelae and their respective disability weights were based on published sources. Vaccination cost was based on international prices lists, presented in 2014 US$.

**Results:**

At an incidence rate of 50 per 100,000/year, routine conjugate vaccination is highly cost-effective in 14 out of 26 countries with a cost/DALY averted ranging from US$555-US$787. At the higher incidence rate of 150 per 100,000/year, quadrivalent vaccination is cost-effective in all 26 countries with a cost/DALY averted ranging from US$105-US$250. The annual incidence rate at which routine conjugate quadrivalent vaccination is expected to be economically justifiable ranges from 13 per 100,000/year in Nigeria to 142 per 100,000/year in Burundi.

**Conclusion:**

Routine quadrivalent conjugate vaccination against *Neisseria meningitidis* is cost-effective at incidence rates well below the epidemic threshold among children living in the African meningitis belt.

## Background

*Neisseria meningitidis* (Nm) or meningococcus is a bacterium that causes meningitis outbreaks that commonly occur in 7–10 year cycles in the African meningitis belt, a region stretching through the African savanna in Senegal in the west to the Red Sea in the east. Epidemics typically occur during dry seasons from December until May, then spontaneously abate at the onset of the rainy season. Approximately 5–10% of the population in the affected region are asymptomatic carriers who may never develop symptomatic disease, but are a potential source of outbreaks [1,2].

The main pathogenic serogroups of Nm are A, B, C, W, X and Y. Clinically, Nm infection presents as meningitis, meningococcaemia (septic shock), arthritis, vasculitis, episcleritis and various neurological sequelae. Following therapy patients may still die, recover with complications, residual disabilities or neurologic deficits, or recover fully [2,3,4]. Major Nm epidemics in the region were primarily due to serogroups A and W, and prior to 1980, serogroup C [2,5,6]. Previously, serogroup C epidemic in the meningitis belt occurred in Nigeria and Burkina Faso in 1975 and 1979 respectively [3,7]. In 1996, the largest meningitis epidemic caused disease in 250,000 persons and 25,000 deaths [5,8]. To contain epidemics, vaccination with polysaccharide vaccines against serogroups A and C was offered to affected communities in the early stages of outbreaks [9,10]. Although less costly, these vaccines are less immunogenic, especially in infants, less efficacious and confer a shorter duration of protection. Furthermore, unlike recently introduced conjugate vaccines, polysaccharide vaccines have no effect on nasopharyngeal Nm carriage.

From 2010 to 2016, a subsidized conjugate meningococcal vaccine against serogroup A (*MenAfriVac*^*®*^), the predominant cause of outbreaks in the region, was administered to over 230 million inhabitants aged 1–29 years in the affected region (Meningitis Vaccine Project; www.meningvax.org). Since then, accumulating evidence confirm that outbreaks due to Nm serogroup A have dramatically reduced [11,12]. However, an emerging concern is that serogroup replacement with C, X, Y and W may occur, and cause future outbreaks. Indeed, in 2014–15, in Niger Republic, an outbreak of serogroup C disease resulted in 9367 cases and 549 deaths and was shown to have caused low level outbreaks in two adjoining states in northwestern Nigeria from late 2013 [13,14,15]. Between December 2016 to June 2017, this blossomed into an epidemic largely due to Nm serogroup C which at its end on 23 June 2017 affected 14,518 cases with 1,166 deaths (a case fatality rate of 8%) in 25 states in Nigeria [16].

Currently, a high proportion of susceptible individuals in the meningitis belt are thought to be protected against serogroup A infection, however the costs and benefits of continuing to vaccinate the same population with vaccines containing other serogroups is unknown. Previous cost-effectiveness and economic evaluations were of a limited nature focusing on polysaccharide vaccines, immunization strategies, meningitis from single serogroup or were conducted for different settings [17,18,19,20].

Therefore, we undertook this cost-effectiveness analysis to determine the optimum approach to prevent emergence and dispersal of common non-A pathogenic serogroups in the meningitis belt by evaluating the cost-effectiveness of routine conjugate quadrivalent vaccination at incidence rates of 50 and 150 cases per 100,000/year and to estimate the annual serogroups C, W and Y incidence rate at which it becomes economically justifiable to broaden coverage from a monovalent to the quadrivalent vaccine.

## Methods

**Fig 1:**
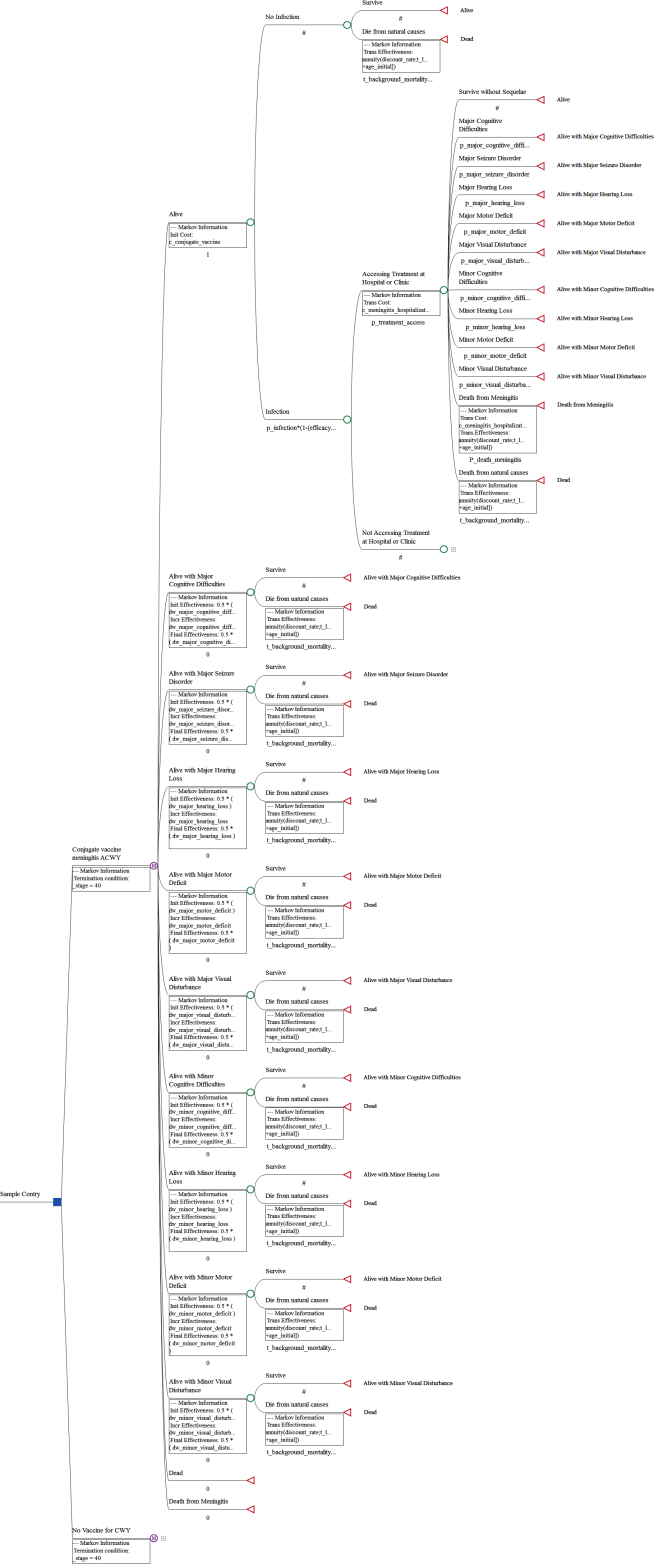
Graphical representation of the cost-effectiveness model structure.

We developed a health economic model from the perspective of the local national health system to evaluate the cost-effectiveness of inter-epidemic routine meningitis with serogroup ACYW conjugate vaccination versus continuing monovalent serogroup A vaccination and customized this model for the 26 countries that lie within the African meningitis belt. Our model was based on a 40-year Markov state transition cohort with annual cycle lengths and the standard half-cycle correction and applied to a cohort of children starting at age 1 to 10. The graphical representation of the model structure is displayed in Fig 1. In any year, children could either be infected with meningitis serogroup CWY or not be infected. The efficacy of the two vaccination strategies in reducing the risk of serotype A was assumed to be the same and therefore not included in the model. Published life tables were used to model the general population risk of mortality among uninfected children. Risk for annual inter-epidemic infection was held constant over time and mirrored incidence thresholds of 1 and 3 infections per week per 100,000 population, which translated into annual incidence risks estimates of 50 and 150 infections per 100,000 population. Although the inter-epidemic rate could be as low as 0.27 cases per week per 100,000 population it is often higher during peaks of seasonal fluctuations and the average of 1 case per week per 100,000 population was used as base-case to model the longer-term cost-effectiveness of the quadrivalent vaccination [12]. Furthermore this incidence rate was selected given it falls well below the revised epidemic threshold of 10 cases per 100,000 population per week [21].

Conjugate vaccines have the highest efficacy in reducing the risk of infection in the first year but efficacy wanes in subsequent years in exponential fashion [20]. Following a non-A meningitis infection, the model first included a chance node describing the likelihood that the infected child would receive medical care at a medical facility and applied a higher mortality rate for cases that do not access medical care. For all surviving cases, the risk of long term sequelae associated with meningitis infection were obtained from a meta-analysis of long-term outcomes following meningococcal infection, adjusted for the higher risk of morbidity associated with the African region [4]. While long-term sequelae were assumed to persist over the remainder of patients’ lifetimes, they were not assumed to have any impact of the risk of mortality. In the model, specific sequelae included major and minor forms of cognitive difficulties, hearing loss, motor deficits and visual disturbance, as well as major seizure disorder. Disability weights corresponding to these outcomes were obtained from the Global Burden of Disease, Injuries, and Risk Factors Study (GBD) [22]. Subsequent infections were not specifically included in the model as it was assumed that all cases of disease transmission were already inherent in the incidence rate.However, for simplicity, additional transmissions beyond the first transmission were not included. Quadrivalent meningitis vaccine cost was based on a World Health Organization (WHO) review of vaccine prices in 2013 [23]. The cost of vaccine administration was assumed to be equivalent in both arms and was therefore not included. Estimated local cost of meningitis hospitalization was obtained from the literature [24]. All long-term health outcomes and costs were discounted at 3% [25]. Results were expressed in terms of the incremental cost-effectiveness ratio (ICER) of the cost/DALY averted. We also estimated the threshold incidence rate at which it is highly cost-effective to routinely vaccinate children with the quadrivalent vaccine in between outbreaks. Calculations were performed using TreeAge Pro 2014, R1.0 (Treeage Software Inc., Williamstown, MA, USA).

### Clinical and cost inputs

Our model inputs are presented in Table 1. In each simulation, the annual risk of non-A meningitis infection was held constant over the full 40-year time horizon in the model at 50 and 150 infections per 100,000 population per year. The first year efficacy of the conjugate vaccine in our model was based on the real world experience associated with a mass vaccination campaign conducted in Chad, which found a 94% reduction in the incidence of meningitis due to serogroup A [11]. We assumed the conjugate vaccine against non-A serogroups has the same efficacy as that against serogroup A. In subsequent years, based on an estimate by an expert panel [13], it was assumed that the average duration of protection would extend to 10 years, which translated into a waning factor of 0.1 (w = 1/average duration of protection). This was used to estimate conjugate vaccine efficacy in a given year as a function of vaccine efficacy in the first year, as well as an exponential function of the waning factor and follow-up time. The follow-up time of 40 years was chosen to account for the full protective effect of the vaccine, which would have disappeared by the end of this period. The probability of treatment access in the event of meningitis infection was based on assessment of patient practices for meningitis infection in northern Ghana, which indicated that over 85% of patients would seek medical care in case of infection [26]. The case fatality rate of 11.7% among treated patients was informed by an assessment of health outcomes among hospitalized patients infected with meningitis A in Niger [27]. Since antibiotic therapy has become the standard of care of meningitis therapy, we based the input describing the mortality rate in the absence of treatment on an international study of health outcomes in meningitis patients that collected data before the availability of antibiotics, which reported a mortality rate of 30.9% [28]. A total of five major and four minor sequelae were listed and presented with their corresponding risk among meningitis patients globally in the meta-analysis by Edmond et al (2010) [4]. The same study reported that patients among the seven studies conducted in the African region faced a 25% risk of at least one major sequelae, compared to a corresponding 13% risk among all 58 included global studies [4]. This ratio of 25%/13% = 1.92 was applied to adjust the reported individual risk estimates of the nine sequelae for the higher risk described in the African region [4]. The footnote in Table 1 describes the specific GBD category that was used to proxy for the disability weights of the nine sequelae that are included in the model. The vaccine acquisition cost was based on a report of vaccine price data published by the WHO for the year 2013 [23], which listed the median procured price per dose of the conjugate meningitis ACWY vaccine at US$ 14.21. The corresponding cost of the monovalent serogroup A vaccine was assumed to cost US$ 0.90 per injection [29], thus yielding a cost differential of US$ 13.31 per dose between the two vaccine options. The cost of meningitis infection was estimated on the basis of a regression model that used local inputs such as per capita GDP or per capita health care expenditures, among others, to estimate the local cost of meningitis treatment in each of the 26 countries included in our model [24].

**Table 1:** Model inputs.

Name	Base case	Range	Distribution	Reference
Annual Meningitis CWY Infection Risk per 100,000 pop.	50, 150	Not varied	n/A	WHO
Conjugate Vaccine Efficacy at Year 1	94.0%	87.2%-98.3%	Beta	Daugla 2014
Conjugate Vaccine Waning Factor	0.1	0.06–0.14	Normal	Maurice 2015
Probability of Treatment Access if Infected	85.1%	79.0%-90.2%	Beta	Hayden 2013
Discount Rate	3.0%	0.0%-6.0%	Beta	WHO-Choice
Outcome Probabilities Associated with Infection				
Death from Meningitis, treated	11.7%	4.6%-20.2%	Beta	Campagne 1999
Death from Meningitis, untreated	30.9%	23.3%-39.0%	Beta	Flexner 1913
Major Cognitive Difficulties^a^	1.3%	0.0%-5.9%	Beta	Edmond 2010
Major Seizure Disorder^a^	1.7%	0.0%-3.8%	Beta	Edmond 2010
Major Hearing Loss^a^	7.3%	2.3%-14.0%	Beta	Edmond 2010
Major Motor Deficit^a^	2.7%	2.1%-7.7%	Beta	Edmond 2010
Major Visual Disturbance^a^	7.1%	4.0%-8.1%	Beta	Edmond 2010
Minor Cognitive Difficulties^a^	13.7%	7.9%-25.6%	Beta	Edmond 2010
Minor Hearing Loss^a^	7.9%	2.1%-9.6%	Beta	Edmond 2010
Minor Motor Deficit^a^	5.8%	5.0%-6.5%	Beta	Edmond 2010
Minor Visual Disturbance^a^	3.5%	2.7%-4.2%	Beta	Edmond 2010
Disability Weights				
Major Cognitive Difficulties^b^	0.126	0.085–0.176	Beta	Salomon 2012
Major Seizure Disorder^c^	0.319	0.211–0.445	Beta	Salomon 2012
Major Hearing Loss^d^	0.032	0.018–0.051	Beta	Salomon 2012
Major Motor Deficit^e^	0.377	0.251–0.518	Beta	Salomon 2012
Major Visual Disturbance^f^	0.191	0.129–0.269	Beta	Salomon 2012
Minor Cognitive Difficulties^g^	0.031	0.018–0.049	Beta	Salomon 2012
Minor Hearing Loss^h^	0.005	0.002–0.012	Beta	Salomon 2012
Minor Motor Deficit^i^	0.012	0.005–0.022	Beta	Salomon 2012
Minor Visual Disturbance^j^	0.004	0.001–0.010	Beta	Salomon 2012
Costs				
Conjugate Quadrivalent Vaccine, per dose	$14.21	Not varied	N/A	WHO
Cost of Monovalent Vaccine, per dose	$0.90	Not varied	N/A	Colombini 2015
Cost of Meningitis Treatment	Country Specific	Country Specific	Normal	Portnoy 2015

^a^ Based on a multiplier of 1.92 for the African region

^b^ Proxied with Intellectual Disability: Severe

^c^ Proxied with Epilepsy: treated, with recent seizures

^d^ Proxied with Hearing Loss: Severe

^e^ Proxied with Motor Impairment: Severe

^f^ Proxied with Distance Vision: Severe Impairment

^g^ Proxied with Intellectual Disability: Mild

^h^ Proxied with Hearing Loss: Mild

^i^ Proxied with Motor Impairment: Mild

^j^ Proxied with Distance Vision: Mild Impairment

### Scenario, threshold and probabilistic sensitivity analyses

We conducted threshold analyses to estimate the incidence rate at which routine conjugate meningitis vaccination is highly cost-effective in a given country, based on established WHO cost-effectiveness thresholds of 1 time per capita GDP [25]. We varied select inputs in probabilistic sensitivity analyses (PSA), whereby 1,000 iterations of the Markov model were run while randomly selecting the values for 28 key model inputs from probability distributions around all efficacy, treatment access, treatment outcomes, disability weights, basic reproduction rate and meningitis treatment cost. The PSA allowed us to estimate the 95% confidence intervals around our ICER estimates and to estimate the proportion of iterations falling below a specific cost-effectiveness threshold.

## Results

Our base-case model results are displayed in Table 2. At an annual incidence of 50/100,000, routine vaccination in children age 1 to 10 is associated with a cost per DALY averted ranging from US$555 in Ghana to US $787 in Central Africa Republic (Table 2). The ICER estimates fall below 1 time per capita GDP for 14 out of 26 countries. In 8 out of the 26 countries in our panel, it is highly likely with a probability exceeding 95% that at an annual CWY incidence rate of 50/100,000, routine quadrivalent vaccination in this age group would be highly cost-effective. Using the higher incidence rate of 150/100,000 results in ICER estimates that all fall below the 1 time per capita GDP threshold, ranging from US$105 in Ghana to $252 in Chad and also increases the likelihood that routine quadrivalent vaccination is cost-effective with a probability exceeding 95% for all countries except Burundi and the Central African Republic.

**Table 2:** Incremental cost effectiveness ratio (ICER) for vaccination in children age 1–10, probability of ICER cost effectiveness with ACWY conjugate vaccine administered for 1 year olds at different annual incidence rates for the 26 meningitis belt countries.

	Per Capita GDP (2015 $)^[^^32^^]^	ICER (95% CI)	Probability (ICER<1 x per capita GDP)	ICER (95% CI)	Probability (ICER<1 x per capita GDP)
Incidence Rate		50/100,000/year	50/100,000/year	150/100,000/year	150/100,000/year
Benin	$860	$645 ($327-$1,117)	81.6%	$185 ($86-$343)	100.0%
Burkina Faso	$640	$695 ($388-$1,201)	45.2%	$229 ($122-$406)	100.0%
Burundi	$260	$741 ($393-$1,359)	0.2%	$245 ($136-$421)	58.5%
Cameroon	$1,320	$703 ($382-$1,240)	98.8%	$207 ($111-$367)	100.0%
Central African Republic	$330	$787 ($451-$1,353)	0.0%	$250 ($144-$435)	79.4%
Chad	$880	$767 ($445-$1,329)	66.1%	$252 ($137-$462)	100.0%
Cote D'Ivoire	$1,420	$725 ($396-$1,237)	99.5%	$207 ($105-$366)	100.0%
DR Congo	$410	$765 ($426-$1,333)	1.5%	$250 ($139-$427)	96.6%
Eritrea	$480	$581 ($281-$1,059)	27.6%	$146 ($61-$287)	100.0%
Ethiopia	$590	$647 ($337-$1,154)	41.7%	$200 ($104-$354)	100.0%
Gambia	$460	$620 ($316-$1,100)	19.5%	$158 ($68-$307)	99.9%
Ghana	$1,480	$555 ($283-$969)	100.0%	$105 ($11-$219)	100.0%
Guinea-Bissau	$590	$718 ($392-$1,302)	24.7%	$225 ($121-$489)	100.0%
Guinea Conakry	$470	$688 ($359-$1,242)	13.0%	$211 ($105-$372)	99.8%
Kenya	$1,340	$655 ($339-$1,184)	98.9%	$186 ($94-$336)	100.0%
Mali	$760	$697 ($388-$1,247)	66.6%	$227 ($123-$396)	100.0%
Mauritania	$1,370	$599 ($319-$1,105)	99.4%	$165 ($76-$324)	100.0%
Niger	$390	$681 ($358-$1,175)	3.6%	$220 ($119-$397)	97.4%
Nigeria	$2,790	$729 ($400-$1,242)	100.0%	$225 ($113-$394)	100.0%
Rwanda	$700	$634 ($326-$1,140)	62.0%	$192 ($96-$345)	100.0%
Senegal	$980	$560 ($279-$1,007)	97.1%	$126 ($43-$266)	100.0%
South Sudan	$790	$711 ($381-$1,272)	64.4%	$221 ($111-$406)	100.0%
Sudan	$1,920	$627 ($328-$1,130)	100.0%	$179 ($87-$328)	100.0%
Tanzania	$920	$647 ($349-$1,126)	89.6%	$173 ($79-$333)	100.0%
Togo	$540	$673 ($367-$1,173)	23.8%	$195 ($99-$359)	100.0%
Uganda	$700	$723 ($385-$1,302)	42.4%	$226 ($128-$401)	100.0%

### Results from threshold analyses

**Fig 2:**
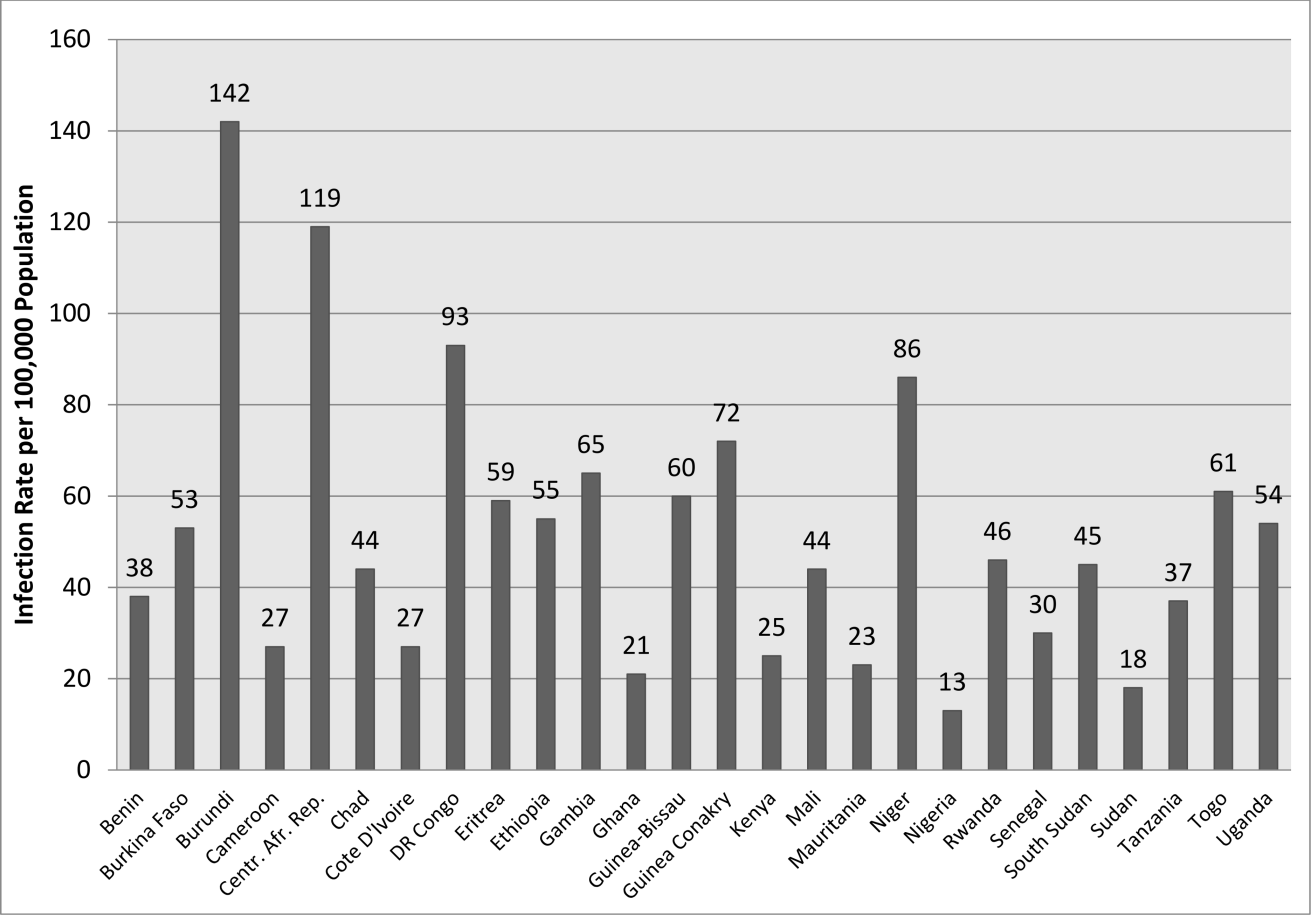
Annual infection rate per 100,000 population at which routine conjugate vaccination is cost-effective in children vaccinated at age 1 to 10.

Fig 2 presents the annual CWY incidence rate at which routine quadrivalent meningitis vaccination would still be highly cost-effective in children aged 1 to 10 among the various countries in our panel. In some higher income countries, such as Nigeria and Sudan, routine quadrivalent vaccination would still be highly cost-effective at annual incidence rates as low as 13-18/100,000. These threshold estimates increase to 119 and 142 for the lowest income countries of the Central African Republic and Burundi. It is only in Nigeria that the estimate falls below the inter-epidemic nadir seasonal incidence rate of 13.5 cases annually (or 0.27 cases per week) per 100,000 population reported by Lingani et al 2015 [12].

## Discussion

This study assessed the cost-effectiveness of broadening meningitis vaccine coverage from serotype A to quadrivalent ACWY in 26 countries in the African meningitis belt using incidence rates that do not currently warrant mass immunization campaigns [21]. We estimate that quadrivalent vaccination would be highly cost-effective at ≥ 95% probability in 24 out of 26 countries at incidence rates corresponding to the current alert threshold of 3 cases per 100,000 per week. These findings suggest that, despite the fairly large difference in the vaccine acquisition cost, for most countries in the African meningitis belt, expanding routine coverage from the monovalent serogroup A vaccine to broader coverage with a quadrivalent ACWY vaccine would provide good value per dollar spent even at incidence rates that fall well below the WHO epidemic threshold and where routine vaccination against the non-A serogroups would currently not be recommended [21].

We also assess the country specific threshold annual incidence rate at which vaccination remains cost-effective. For Nigeria, if the annual non-A incidence is as low as 13 per 100,000 (0.26 cases per 100,000 weekly) the estimate remains highly cost-effective while in Central Africa Republic and Burundi annual incidence must be at least 119 and 142 per 100,000 respectively (around 2–3 cases per 100,000 weekly) to remain highly cost-effective. In Nigeria, even at the background nadir of 0.27 cases per week or 13.5 cases annually per 100,000 population, expanding coverage of the quadrivalent vaccine is likely to remain highly cost-effective even if there is never an outbreak over the next 40 years (Fig 2). For remaining countries, the threshold falls somewhere in between 20 to 100 per 100,000 annually (0.4 to 2 cases per 100,000 weekly), which is well below the WHO defined Alert Threshold of 3 cases per 100,000 weekly [21]. Notably, the Alert Threshold is used for public health containment intervention measures during or leading up to meningitis outbreaks, whereas our model assumed a constant incidence rate over the 40 year time horizon. However, our results suggest that quadrivalent vaccination constitutes a worthwhile healthcare investment as long as a relatively low non-A serogroup incidence rate is observed and expected to persist in the general population.

We provide estimated costs per DALY averted when vaccination is administered for children aged from 1 year and from10 years because routine immunization could potentially be administered as part of school health programmes as previously suggested [17,18]. Price reductions in vaccines against multiple serogroups to below $1 per dose (current price of vaccines against serogroup A), would result in estimates where the quadrivalent vaccine dominates the monovalent one, as a similarly priced quadrivalent vaccine would reduce the risk of transmission of non-A serogroups, resulting in fewer meningitis related treatment costs, while also averting the DALYs resulting from non-A infections. This argues strongly for a similar concerted approach to contain epidemic meningitis due to non-A serogroups.

Non-A serogroups (C, W, Y, others) are an important contributor to outbreaks in the meningitis belt [3,6,13,14,15,21,30]. However, they can cause disease of similar severity and outbreak of similar magnitude as those caused by serogroups A [3,6,15]. Consequently, the favorable cost-effectiveness analysis estimates of routine immunization with quadrivalent conjugate vaccine warrants consideration of the intervention as an international public health priority for the region.

A previous study reported a cost per QALY gained of $199 for a combination of routine and mass vaccinations versus mass vaccination alone at the onset of meningitis epidemics due to *Neisseria meningitidis* [17]. However, the base-case incidence rate of 250 per 100,000 was two to five times the base-case used in our study. From our scenario analysis, that incidence rate would result in lower ICER estimates ranging from $15 to $149 per DALY for the 26 countries, lower than their estimate [17].

The study has certain limitations. Firstly, meningococcaemia (or sepsis) resulting in shock was not captured as a separate entity in the model largely because mortality estimates of meningitis during epidemics often encompasses both meningitis and shock. As disaggregated data are not available, mortality estimates for meningitis generally represent the weighted average of the two entities. Secondly, we assumed about 85% of the population access either hospital or clinic-based healthcare during epidemics as observed in Ghana but data from other countries was not available [25]. Thirdly, the analysis neither incorporated age-specific *Neisseria meningitidis* transmission pattern nor its seasonality in the annual incidence rates but it is likely that this evens out. Fourthly, quadrivalent vaccine does not cover some pathogenic sero-groups such as X which has already caused meningitis outbreaks in Niger [31]. In future such sero-groups may replace vaccine sero-groups as a main cause of outbreaks. Lasting solutions to sero-group replacement are needed and experimental Nm DNA vaccines may prove useful in this regard. Lastly, following introduction of routine immunization, it is probable meningitis conjugate vaccine will confer herd immunity by virtue of significant reduction in carriage and high vaccine population coverage may no longer be needed to attain good protection. These effects were not included in the analysis, although it stands to reason that fully incorporating the effect of herd immunity would merely decrease the ICER estimates further and therefore have a limited impact on the conclusion of this study.

## Conclusion

Expanding the coverage of routine immunization against *Neisseria meningitides* from a monovalent serogroup A to a quadrivalent ACWY vaccine is cost-effective at incidence rates below the epidemic threshold among children living in the African meningitis belt. It should be considered an international public health priority.
